# Development and Application of the Chinese (Mainland) Version of Chronic Liver Disease Questionnaire to Assess the Health-Related Quality of Life (HRQoL) in Patients with Chronic Hepatitis B

**DOI:** 10.1371/journal.pone.0162763

**Published:** 2016-09-15

**Authors:** Yanli Liu, Sai Zhang, Yali Zhao, Juan Du, Guanghui Jin, Shuang Shao, Xiaoqin Lu

**Affiliations:** Department of General Practice, School of General Practice & Continuing Education, Capital Medical University, Beijing 100069, China; Chang Gung Memorial Hospital Kaohsiung Branch, TAIWAN

## Abstract

**Objective:**

To develop the Chinese (Mainland) version of Chronic Liver Disease Questionnaire (CLDQ) and use it to assess the health-related quality of life (HRQoL) of chronic hepatitis B (CHB) patients in China and identify the determinants of HRQoL.

**Methods:**

The Chinese (Mainland) CLDQ was developed by expert consultation, focus group interviews with patients, and pilot study. The final version of questionnaire was adopted to assess the HRQoL of chronic hepatitis B outpatients enrolled from two largest infectious hospitals in Beijing. Cronbach’ s alpha was used to measure the internal consistency reliability. The construct validity was measured by factor analysis. T-test, one-way analysis of variance (ANOVA), and multi-variable linear regression were used to analyze the data.

**Results:**

Cronbach’s alpha of the overall CLDQ is 0.935, ranging from 0.628 to o.881 among six subscales. Six factors were identified via factor analysis, including a new factor sleeping(SL). A total of 519 patients with CHB were included in the investigation with the final version of questionnaire, 405 of them were only with CHB, 53 with compensated cirrhosis, and 61 with decompensated cirrhosis. The CHB group scored the highest in the overall score of CLDQ (p<0.05). The score of worry (WO) domain was significantly lower in the compensated group than the CHB group (p<0.05). Decompensated cirrhosis patients scored lower than the CHB group in all CLDQ domains and the overall score (p<0.05). Stages of illness, gender, regular visits to specialized hospitals, and work status in last year were determinants of HRQoL.

**Conclusion:**

The psychometric properties of the Chinese(Mainland) CLDQ is acceptable. The HRQoL of CHB patients deteriorated with disease progression. Advanced stages of CHB, female, long time absence from work after illness, and no job or retirement were determinants of poor HRQoL. Regular visits to specialized hospitals was a positive determinant of HRQoL.

## Introduction

Viral Hepatitis has been a major global health problem. More than 240 million people world-wide have chronic hepatitis B infections. And more than 780,000 people die every year due to acute or chronic consequences of hepatitis B [1]. Thirty percent chronic HBV infectious patients develop into CHB and 12%-15% CHB patients develop into cirrhosis within 5 years [2]. Patients with CHB are at high risk developing into decompensated cirrhosis and hepatocellular carcinoma (HCC) [3]. The mortality of CHB, compensated cirrhosis and decompensated cirrhosis within 5 years are 0~2%, 14%~20%, 70%~86%, respectively [4]. China has the highest carrier prevalence of HBV infection in the world, accounting for 10% of the general population [5]. In China, CHB related-disease posed substantial threat on patients’ health and resulted in heavy economic burden on their families and the society [6,7].

In recent years, increasing attention has been focused on patients’ quality of life. Health-related quality of life (HRQoL), a measurement of physiological, psychological, and social dimensions, has been an important indicator frequently used in chronic disease in the last two decades. Chronic Liver Diseases Questionnaire (CLDQ) is a disease-specific HRQoL instrument which was developed by Younossi firstly [8]. It consists of 29 items divided into 6 dimensions including fatigue (FA), activity (AC), emotional function (EF), abdominal symptoms (AS), systemic symptoms (SS), and worry (WO). The CLDQ scores of each item range from 1 to 7, indicating “all of the time” to “none of the time”, respectively. The overall score is calculated by the mean scores of all subscales. The CLDQ has been translated into different languages and was mainly used in HCV infectious patients [9–11]. There is few study on its application in Chinese CHB patients.

The Chinese (Hong Kong, HK) CLDQ was developed by Elegance et al. and was proved to have acceptable reliability, validity and sensitivity in measuring the HRQoL of CHB patients [12]. However, participants enrolled in Elegance’ study were only those who can speak Cantonese, a dialect completely different from Mandarin. Furthermore, the economic status and cultural background between HK and Mainland China are significantly different. Therefore, some items of Chinese (HK) CLDQ may be not suitable for Mainland China.

The aim of this study was to develop a Chinese (Mainland) version of CLDQ based on original CLDQ and evaluate the HRQoL of patients at different stages of chronic hepatitis B in Mainland China. We also analyzed the determinants of HRQoL in CHB patients.

## Methods

### Ethics statement

The study obtained ethics approval from Ethics Board of Center for Disease Control and Prevention, Beijing, China. Our investigators informed the participants the purpose of this study with ensured confidentiality. All participants signed the informed consent and were aware that they can withdraw from this study at any time for any reason. The consent procedure was approved by the ethics committee.

### Development of the Chinese(Mainland) version of CLDQ

The original CLDQ was translated into Chinese respectively by a clinical chronic hepatitis B expert and a clinical psychology expert with translation experience. Revision of the rhetoric and expression was carried out by another psychology expert in HRQoL measurement and a bilingual translator. The Chinese CLDQ was back-translated into English by two other bilingual translators. The difference between the back translated CLDQ and the original CLDQ was compared by two bilingual experts in chronic hepatitis B related work to further revise the rhetoric and expression of the Chinese(Mainland) CLDQ. The first draft of the Chinese(Mainland) CLDQ was completed by 10 selected patients with CHB and then evaluated by them through focus group interviews to figure out the problems on syntax and semantics. The second revision of the Chinese(Mainland) CLDQ was made on the basis of the focus group interviews and experts consultation.

Sixty patients with CHB were recruited to participate in the pilot study. The inclusion criteria included patients aged 18 or above, living in Beijing for 6 months or above, documented diagnosis of CHB or CHB related cirrhosis. The diagnosis of CHB is based on the presence of hepatitis B surface antigen and/or HBV DNA for more than six months, with increased serum alanine aminotransferase. The diagnosis of compensated cirrhosis is based on the liver biopsy or imaging findings of ultrasound or computed tomography (CT). The decompensated cirrhosis is defined as having serious complications of cirrhosis, including ascites, variceal bleeding, or hepatic encephalopathy. The detail information about diagnostic standards and classification of CHB referred to the Guideline of Prevention and Treatment for Chronic Hepatitis B (China, 2010 Version) [13] issued by the Society of Infectious Diseases, China Medical Association (CMA). The following patients were excluded: (1) currently diagnosed with other chronic diseases, (2) co-infected with diseases such as hepatitis C virus (HCV), hepatitis D virus (HDV) or human immunodeficiency virus (HIV), (3) severe psychiatric conditions, (4) chemical dependency including heavy episodic drinking defined as a consumption of 60 or more grams of pure alcohol on at least one single occasion at least monthly or illegal drug, and (5) pregnant or unable to communicate with others. After the pilot study, in- depth interviews were conducted with patients who had difficulties in completing the questionnaire. Eventually, the third amendment was carried out to form the final version of the Chinese (Mainland) CLDQ.

### Assessment of HRQoL using the Chinese(Mainland) CLDQ

To test the psychometrics of the Chinese (Mainland) CLDQ and to identify the determinants of HRQoL in CHB patients, a cross-sectional study was conducted from April 2011 to March 2012 in Beijing, China. All participants were outpatients selected from Ditan Hospital and Youan Hospital, two largest specialized hospitals of infectious diseases in Beijing. The inclusion and exclusion criteria were described above. All participants were asked to complete the Chinese (Mainland) CLDQ and provide socio-demographic and clinical information(refer to S1 File). The investigators involved in this study were trained before the investigation.

All participants were divided into CHB group (without cirrhosis), compensated cirrhosis group and decompensated cirrhosis group according to the Guideline of Prevention and Treatment for Chronic Hepatitis B (China, 2010 Version).

### Statistical analysis

EpiData3.0 was used to establish the database and double-entry was performed by two persons to avoid errors. All data analyses were carried out in SPSS for Windows 17.0. Socio-demographic and clinical information were analyzed using t- test, one-way analysis of variance (ANOVA) or rank sum test. Statistical significance levels were set at p value less than 0.05. ANOVA was used to compare the difference among three patient groups. Cronbach’s alpha was employed to measure the internal consistency reliability of the questionnaire and factor analysis was used to measure the construct validity of the Chinese CLDQ. Multi-variable linear regression was performed to identify the determinants of HRQoL in CHB patients, with p value at 0.10.

## Results

### The Chinese (mainland) CLDQ

After the pilot study, sixty patients indicated that the questionnaire was easy to understand and could be completed within 10 minutes. The revision focused on the rhetoric and expression of 8 items (N7, N13, N14, N19, N21, N22, N25, N28) which did not meet their expression habits. Item N29 “how much of the time during the last 2 weeks have you been concerned about the availability of a liver if you need a liver transplant” was revised for the most. Fifty-three (88.3%) participants indicated this item was not applicable, because the majority of CHB patients in Mainland China had little chance of receiving liver transplant due to economic reasons. Thus, according to the suggestion of participants, N29 was revised into “in the past 2 weeks, how much time have you worried that your economic reasons will impact on your disease treatment?”. Please see S2 File for the original CLDQ and the back-translated Chinese(Mainland) CLDQ.

### Socio-demographic and clinical information of participants and overall scores of CLDQ among different groups

A total of 630 patients were included to participate in this survey and 95 patients with other chronic diseases were excluded. Sixteen questionnaires were invalid. A total of 519 questionnaires were valid in the end. The mean age was 38.8±12.0 years old (range: 18–81 years) and 58.4% of the patients were male. Among the 519 participants, 405 (78.1%) were CHB patients, 53 (10.2%) were patients with compensated cirrhosis, and 61(11.8%) were patients with decompensated cirrhosis. The socio-demographic and clinical information of the three patient groups are elaborated in table 1.

**Table 1:** Social-demographic and clinical information of the participates.

	CHB (n = 405)	Compensated cirrhosis(n = 53)	Decompensated cirrhosis(n = 61)	Overall (n = 519)
Age, yr (mean ± SD)	36.1±10.7	49.5±11.3	46.9±11.6	38.8±12.0
Gender (%)				
Male	55.3	75.4	60.4	58.4
Female	44.7	24.6	39.6	41.6
Education level (%)				
Primary or below	5.9	13.1	11.3	7.3
Junior or equivalent	27.9	36.1	32.1	29.3
High school or equivalent	23.2	27.9	34	24.9
University or above	43	23	22.6	38.5
Types of Medical Insurance (%)				
Self-expense	41.7	13.1	30.2	37.2
Others	58.3	86.9	69.8	62.8
Marital status (%)				
Married	80	86.9	92.5	82.1
Others	20	13.1	7.5	17.9
Duration of illness, yr (mean ± SD)	7.9±7.9	10.5±9.7	10.9±10.4	8.5±8.4
Work status in last year (%)				
Retirement or no job	20.2	57.4	49.1	27.6
Sick leave for 6 months or above	1.2	3.3	1.9	1.5
Sick leave for 3 to less than 6 months	5.4	11.3	3.8	6.2
Keep working or sick leave for less than 3 months	73.1	26.2	45.3	64.7
Drinking in last month				
Yes	9.4	4.9	1.9	8.1
No	90.6	95.1	98.1	91.9
Family history of CHB				
Yes	32.3	36.1	32.1	32.8
No	61.2	59	66	61.5
Unclear	6.4	4.9	1.9	5.8
Regular visits to specialized hospitals				
Yes	68.9	67.2	77.4	69.6
No	31.1	32.8	22.6	30.4
Anti-viral treatment				
Yes	68.4	86.9	79.2	71.7
No	29.6	13.1	17	26.4
Unclear	2	0	3.8	1.9
Expense for CHB in last year (%)				
≤1000	15.3	18	11.3	15.2
1001~5000	21.2	29.5	30.2	23.1
5001~10000	16.8	11.5	5.7	15
10001~20000	21.5	16.4	32.1	20
>20000	25.2	24.6	20.8	24.7
Annual per capita Household income (%)				
<10000	34.6	42.6	41.5	36.2
10000–19999	14.3	19.7	20.8	15.6
20000–29999	16.8	18	26.4	17.9
>30000	34.3	19.7	11.3	30.3

### CLDQ scores in different stages of CHB

The overall CLDQ score was 5.2±1.0 and the mean scores of 6 domains including abdominal symptom (AS), fatigue (FA), systemic symptoms (SS), activity (AC), emotional function (EF), and worry (WO) were 5.8±1.3, 4.9±1.3, 5.6±1.0, 5.7±1.3, 5.2±1.2, 4.7±1.4, respectively.

ANOVA was used to compare the difference among three patient groups and significant difference was seen in all domains of CLDQ (p<0.05). CLDQ scores by patient groups are shown in Table 2. The CHB group scored the highest in overall score among the three patient groups (p<0.05). The score of WO domain was significantly lower in the compensated group than the CHB group (p<0.05). The decompensated cirrhosis group scored lower in all domains than the compensated cirrhosis group, but all of the differences did not reach statistical significance except for the SS domain and the overall score (p<0.05). However, the decompensated cirrhosis group scored lower than the CHB group in all CLDQ domains and the overall score(p<0.05).

**Table 2:** CLDQ scores in different stages of CHB.

Domains	CHB(n = 405)	Compensated cirrhosis(n = 53)	Decompensated cirrhosis(n = 61)	F	P
AS	5.9±1.2***	5.5±1.3	5.0±1.6*	17.183	0.000
FA	5.0±1.2***	4.7±1.2	4.5±1.3*	4.718	0.009
SS	5.8±0.9***	5.5±0.9***	4.9±1.0*^,^**	22.199	0.000
AC	5.7±1.2***	5.6±1.1	5.1±1.6*	7.093	0.001
EF	5.3±1.1***	5.2±1.0	4.8±1.3*	5.485	0.004
WO	4.9±1.4**^,^***	4.3±1.4*	4.0±1.4*	14.824	0.000
Overall	5.4±0.9**^,^***	5.1±0.9*^,^***	4.7±1.1*^,^**	15.834	0.000

Note: AS, abdominal symptoms; FA, fatigue; SS, systemic symptoms; AC, activity; EF, emotional function; WO, worry.

*P<0.05 compared with the CHB group.

**P<0.05 compared with the compensated cirrhosis group.

***P<0.05 compared with the decompensated cirrhosis group.

### The psychometrics of the Chinese (mainland) CLDQ

The cronbach’s α of total CLDQ was 0.935, ranging from 0.628 to 0.881 among six subscales. Factor analysis identified 6 factors, which explained 62.68% of the total variance. The items of FA and SS loaded on the same factor(factor 1), except for item “decreased strength” and “bodily pain” which loaded on WO and AS, respectively. Different from the structure of the original questionnaire, the factor of SL was extracted from the 29 items, which included “difficulty in sleeping at night”, “difficulty in falling asleep at night”, and “problems with concentration”. The item “trouble in lifting or carrying heavy objects” was loaded on factor 1. Detailed information on psychometric properties of the Chinese (mainland) CLDQ is shown in table 3.

**Table 3:** The psychometrics of the Chinese(Mainland) CLDQ.

Items	Cronbach’s α	Factor 1	Factor 2	Factor 3	Factor 4	Factor 5	Factor 6
		FA+SS	WO	EF	AS	SL^!^	AC
Abdominal symptoms	0.796						
1:Abdominal bloating		0.135	0.132	0.163	0.725*	0.138	0.124
5:Abdominal pain		0.134	0.093	0.097	0.840*	0.065	0.042
17:Abdominal discomfort		0.165	0.278	0.182	0.726*	0.090	0.106
Fatigue	0.834						
2:Tiredness or fatigue		0.663*	0.139	0.174	0.264	0.170	0.125
4:Feel sleepy during the day		0.766*	0.090	0.187	0.061	0.036	-0.031
8:Decreased strength		0.414	0.495*	0.155	0.232	0.057	0.195
11:Decreased energy		0.637*	0.235	0.260	0.224	0.168	0.188
13:Drowsiness		0.755*	0.109	0.288	0.010	0.010	0.068
Systemic symptoms	0.628						
3:Bodily pain		0.459	0.152	0.012	0.591*	0.041	0.023
6:Shortness of breath		0.505*	0.181	0.085	0.246	0.230	0.209
21:Muscle cramps		0.254*	0.313	-0.082	0.057	0.250	0.103
23:Dry mouth		0.379*	0.281	0.214	0.138	0.278	-0.090
27:Itching		0.333*	0.219	0.150	0.117	0.211	-0.398
Activity	0.744						
7:Loss of appetite		0.301	0.141	0.192	0.222	0.121	0.739*
9:Trouble in lifting or carrying heavy objects		0.659*	0.190	0.135	0.236	0.155	0.114
14:Bothered by loss of appetite		0.254	0.206	0.348	0.210	0.134	0.700*
Emotional function	0.881						
10:Anxiety		0.286	0.216	0.727*	0.125	0.110	0.089
12:Unhappiness		0.197	0.204	0.784*	0.055	0.122	0.085
15:Irritability		0.147	0.183	0.745*	0.156	0.128	0.004
16:Difficulty in sleeping at night		0.133	0.057	0.278	0.132	0.837*	-0.011
19:Mood swings		0.160	0.181	0.735*	0.169	0.226	0.139
20:Difficulty in falling asleep at night		0.166	0.044	0.208	0.122	0.841*	0.084
24:Depression		0.182	0.302	0.712*	0.081	0.130	0.099
26:Problems with concentration		0.350	0.285	0.370	0.016	0.407*	0.196
Worry	0.866						
18:Worries about the impact of the liver disease on family		0.226	0.655*	0.238	0.102	-0.071	0.074
22:Worries that symptoms will develop into major problem		0.075	0.799*	0.162	0.163	0.129	0.092
25:Worries that the condition is getting worse		0.109	0.828*	0.233	0.130	0.083	0.059
28:Worries about never feeling any better		0.137	0.830*	0.204	0.094	0.096	0.037
29:Worries that the economic will effect on treatment		0.154	0.590*	0.235	0.130	0.026	-0.078
Percent of eigenvalue		36.28%	7.05%	6.49%	4.93%	4.34%	3.59%
Cronbach’s α of the whole questionnaire	0.935						

AS, abdominal symptoms; FA, fatigue; SS, systemic symptoms; AC, activity; EF, emotional function; WO, worry; SL, sleep.

*:The highest value of factor loading.

### The determinants of HRQoL in CHB patients

Table 4 showed the results of multi-variable linear stepwise regression. Stages of illness, work status in last year, education levels, antivirus treatment, family history of HBV infection, expense for chronic hepatitis B in last year, and annual per capita household income were entered as independent variables. Gender, insurance types, marital status, and regular visits to specialized hospitals were entered as dichotomous variables. Age and duration of illness were treated as continuous variables. This model showed that stages of illness, regular visits to specialized hospitals, work status in last year, and gender were determinants of the HRQoL in CHB patients.

**Table 4:** Multi-variable linear stepwise regression on CLDQ overall scores in CHB patients.

	Unstandardized Coefficient(95% CI)	Standardized Coefficient	t	p
Stages of illness (vs. CHB)				
Compensated cirrhosis	-0.646(-0.907,-0.385)	-0.217	-4.854	0.000
Decompensated cirrhosis	-0.262(-0.532,-0.007)	-0.083	-1.911	0.057
Regular visits to specialized hospitals (vs. no regular visits to specialized hospitals)	0.296(0.123,0.469)	0.142	3.370	0.001
Work status in last year (vs. keep working or sick leave for less than 3 months)				
Sick leave for 6 months or above	-0.858(-1.505, -0.210)	-0.110	-2.603	0.010
No job or retirement	-0.177 (-0.366, -0.013)	-0.082	-1.834	0.067
Gender, male (vs. female)	0.0.173(0.009,0.338)	0.089	2.070	0.039
Constant	5.105(4.918,5.293)	-	53.540	0.000

Note: R square of this model is 0.103.

## Discussion

China has the largest CHB population in the world, with 20 to 30 million patients suffering from CHB [14]. However, only a few studies had focused on the HRQoL of CHB patients in Mainland China. Ditan Hospital and Youan Hospital are two largest specialized hospitals of infectious diseases in Beijing and can provide adequate samples for our study. Therefore, we selected this two hospitals.

In this study, item 29 “how much of the time during the last 2 weeks have you been concerned about the availability of a liver if you need a liver transplant?” in the original CLDQ was revised into “in the past 2 weeks, how much time have you worried that your economic reasons will impact on your disease treatment?”in the Chinese (Mainland) CLDQ. However, the meaning of item 29 in Chinese(HK) CLDQ was similar to the original CLDQ. It may be the reason that Mainland China is less developed than Hong Kong in economic level and medical insurance system, which make patients in Mainland China more concern about economic problems. In addition, 8 items (N7, N13, N14, N19, N21, N22, N25, N28) were different from the original CLDQ and 7 items(N6, N13, N14, N15, N19, N20, N28) were different from the Chinese(HK) CLDQ. It indicated that the expression of the Chinese(Mainland) CLDQ established in this study may be more likely to be applicable for patients speaking Mandarin in Mainland China.

This study found that there was significant difference among three groups in the overall score, the CHB group scored the highest while the decompensated group scored the lowest, which indicated that HRQoL of CHB patients deteriorated with disease progression. The score of WO domain was significantly lower in compensated group than patients with HBV only, which meant patients with compensated cirrhosis were more worried about liver disease. The study of Hong Kong also reported a similar findin [7]. It might be explained that patients with compensated cirrhosis were more concerned about decompensation and HCCs in the future. The results also showed that the score of SS domain was significantly lower in the decompensated cirrhosis group than the compensated cirrhosis group. This finding was similar to the result of another study from Xi’an province, China [12]. It can be interpreted that the HRQoL in patients with decompensated cirrhosis was more likely to be affected by the symptoms of complications including ascites, variceal bleeding or hepatic encephalopathy than compensated cirrhosis patients.

In this study, the Chinese (Mainland) CLDQ has acceptable reliability and validity. The factor structure of the Chinese(Mainland) CLDQ was almost identical with the original CLDQ in WO, EF, AS and AC domains. Different from the original CLDQ, a new factor SL was extracted from the questionnaire, including item 16, 20 and 26. This result is similar with that of Chinese (HK) CLDQ, except for item 26 “problems with concentration”. It may be the reason that problems besides emotional function may lead to sleep difficulties as well and might cause concentration problems. The item “trouble in lifting or carrying heavy objects” was loaded on FA and SS, indicating that this item may reflect FA. The item “body pain” was loaded on AS, which may suggest that the symptom of pain in CHB patients mainly located in abdomen.

Previous studies had shown that progression of CHB was one of the determinant of poor HRQoL [15], which was also proved in our study. With progression of liver dysfunction, patients with advanced CHB will suffer from more serious impairment on both physical and mental function. What’s more, advanced stage of CHB will lead to serious complications. Therefore, it is important for CHB patients to initiate regular tests and appropriate treatment even in early stages without symptoms. However, a study showed that 84% investigated CHB patients who were potential candidates for antiviral therapy had not been treated [16]. One reason might be the wrong perception of the necessity of treatment when the symptoms were absent in early stages. Thus, health education and detailed explanation from clinicians are needed and it is necessary for screening and treatment even in early stages without symptoms.

Female was a negative determinant of HRQoL in our study, as was in other studies [17,18]. The finding indicates that gender should be considered when it comes to HRQoL improvement.

Sick leave for more than 6 months and no job or retirement were determinants of poor HRQoL in CHB patients. These can be explained in two main aspects. First, long time absence from work may be related to more serious illness. HRQoL deteriorates with disease progression. Second, no job or retirement because of illness, in conjunction with the heavy economic burden, may increase the sense of worthlessness in these patients and may have negative effect on their HRQoL. Moreover, patients with chronic HBV infection have barriers in working ability compared with healthy workers, which may reduce their household income further.

Regular visits to specialized hospitals within 6 months was positive determinant of HRQoL in CHB patients, which is a new finding compared with previous studies [12,17]. It inferred that patients’ compliance is an important influencing factor on HRQoL of CHB patients. The European [19] and Asian Pacific Associations [4] for the Liver (EASL and APASL, respectively) all emphasized that patients with chronic hepatitis B infection have to be followed up every 3 to 12 months given the severity of disease. However, asymptomatic patients may ignore the development of the disease and the importance of follow-up. A study from Singapore [20] revealed that 84 out of 192 (44%) investigated patients with CHB had not been followed up for over 12 months. Forgetting the date of screening and waiting for a long time for taking blood were two reasons related to the compliance of patients in their study. Therefore, streamlining the process of venipuncture and providing reminder to patients for the date of follow-up were recommended in that study. Management of CHB including health education and regular tests also should be improved in these patients.

Furthermore, comparing with other hospitals, visiting specialized hospitals for infectious diseases may be beneficial for improving the HRQoL of CHB patients. Advanced equipments, high quality of diagnosis and treatment in these hospitals may be the main reasons. However, the number of specialized hospitals in China is small and the medical staffs specialized in infectious liver diseases are scarce, which results in a large number of patients can’t receive treatment in these hospitals. Heavy economic burden of disease treatment is another barrier for CHB patients attending specialized hospitals. Thus, the management of CHB in primary care is increasingly important. As the “gatekeepers” of the health care system, general practitioners (GPs) play a crucial role in the management of chronic disease. In the care of patients with CHB, GPs can do screening at community level and refer the patients to specialists as necessary. Moreover, GPs can assist specialized hospitals to monitor the progression of CHB and provide appropriate health education to the patients and their family members. However, there exist some problems in the management of CHB in primary care. A study from Australia showed that inadequate knowledge of GPs, poor communication with specialists, and health system restrictions are major challenges for the management of CHB by GPs in primary care, which are similar to the situation in China [21,22]. Therefore, Chinese government should increase the funding to resolve these problems. On the one hand, financial support should be increased for GP training and testing equipments deploying. On the other hand, government should also provide public health intervention including community-based screening, health education, and appropriate treatment.

Our study has some limitations. First, the sample size in our study is smaller than expectation, we excluded all co-morbidity patients. Second, all participates were enrolled from two hospitals in one city. The results can't represent other areas in China. Moreover, all participates were from tertiary hospitals, so hospitalization rate bias may exist in this study. However, there exist some problems in the management of CHB in primary care of China, for instance, shortage of sufficient knowledge about HBV and HBV-related diseases as well as lack of examination equipment and antiviral drugs [21], thus, most patients with CHB are still managed in tertiary hospitals in China.

In addition, limited by research funding, we did not include healthy people as a negative control group in this study. Further study will be conducted to compare the HRQoL of patients with chronic HBV infection with healthy people using this CLDQ.

## Conclusions

This study had developed a Chinese (Mainland) CLDQ with acceptable reliability and validity when used in CHB patients. The results of cross-sectional study showed that advanced stages of CHB, female, long time absence from work after illness, and no job or retirement were determinants of poor HRQoL. Regular visits to specialized hospitals was a positive determinant of HRQoL.

## Supporting Information

S1 File The questionnaires used in this study (Chinese and English Versions). (ZIP)

S2 File The original CLDQ and the Chinese (Mainland) CLDQ. (DOCX)
