# Supplementary material for: Development and Application of the Chinese (Mainland) Version of Chronic Liver Disease Questionnaire to Assess the Health-Related Quality of Life (HRQoL) in Patients with Chronic Hepatitis B
Source: PLoS One. 2016 Sep 15;11(9):e0162763. doi: 10.1371/journal.pone.0162763 (PMC5025145; doi:10.1371/journal.pone.0162763)
Supplement: S1 File — (ZIP) [file pone.0162763.s001.zip › CLDQú¿Mainlandú⌐questionnaires.docx]

**Chronic Liver Disease Questionnaire** Visiting Cards No.□□□□□□

Dear patients,

In order to provide better services for patients and improve the quality of medical services, we will conduct an survey on quality of life and disease situation for chronic hepatitis B patients in outpatient of Ditan Hospital and Youan Hospital. The survey is anonymous.The information you filled is only used to research and to improve the quality of service and your personal information will not be exposed. Please tick "√" on your selected answer number. Thank you very much for your support and cooperation.

Capital Medical University

April 8, 2011

**I. Basic Information on Patients**

N1 Your gender is：

① male ② female

N2 Your age is： years old.

N3 Your education level is：

① University and above ② High school and equivalent ③ Junior middle school

④ Primary school ⑤ Illiteracy

N4 Your occupation is:

① Professional and technical personnel ② Workers ③ Agricultural laborers

④ Students ⑤ Retired ⑥ Housework or unemployed ⑦ Business Services ⑧Others

N5 The type of your medical insurance is:

① Social health insurance ② Public expense ③ New rural cooperative

④ Own expense ⑤ Commercial medical insurance ⑥ Others

N6 Your marital status is：

① In marriage ② Single ③ Divorced or widowed

N7 When were you diagnosed with hepatitis B (specific to month)?

( How many years in total? years )

N8 Are you diagnosed with cirrhosis now?

①No ②Yes （If yes, the time of diagnosis(specific to the month)is ）

N9 Have you been told by the doctor that you suffered from complications of liver cirrhosis since the illness?

①Yes ②No**（Skip to N11）**

N10 Which types of disease is your complications of cirrhosis belonging to? （Multiple choice）

①Ascitic（① Regression within 4 weeks ② Regression more than 4 weeks or persist）

②Abdominal infections (such as peritonitis, biliary tract infections, etc.)

③Upper gastrointestinal bleeding

④Hepatic encephalopathy（①PhaseⅠ ②PhaseⅡ ③Phase Ⅲ ④Phase Ⅳ）

⑤ Electrolyte disorders ⑥Others

N11 What types of your disease stages belonging to?

①Chronic Hepatitis B（a Resting b Activity）The duration is months

②Chronic hepatitis B with cirrhosis（a Compensated b Decompensated） The duration is months

③Hepatic carcinoma The duration is months

N12 Did you drink in last month?

①Yes ②No

N13 Other chronic diseases you are suffering from include:

① Diabetes ② Hypertension ③ Coronary Heart Disease ④ Stroke

⑤ Osteoporosis ⑥ Others ⑦ No chronic diseases

N14 After illness, did you regularly visit to specialized hospitals?

①Yes ②No

N15 Have you ever used antiviral therapy since the illness?

①Yes ②No ③ Unclear

N16 In last year,how about your work status?

① Persist working ② Sick leave for 3 to 6 months ③ Sick leave for more than 6 months

④ Haven’t work since illness ⑤ Retired

N17 In last year, how much was your medical expenses in total due to chronic liver disease (excluding transportation costs)?

① Less than 1,000 yuan ② 1,000 to 2,000 yuan ③ 2,000 to 3,000 yuan ④ 4,000 to 5,000 yuan

⑤ 5,000 to 10,000 yuan ⑥ 10,000 to 20,000 yuan ⑦ Above 20,000 yuan

N18 How much is your total household income in last year? yuan. How much is the per capita household income in last year? yuan per person.

N19 Does anyone in your family has hepatitis?

① Yes ② No ③ Unclear

N20 In last year, have you gone to see community physicians/general practitioners due to chronic liver disease?

① Yes ② No

N21 If the community physicians/general practitioners are available to provide follow-up service for chronic hepatitis B, are you willing to accept this service?

① Yes ② No

**II. Assessment of quality of life**

The purpose of this section is to understand how you felt in the past 2 wk. Please answer all questions. You can only choose one answer for each question.

1. In the past 2 wk, how much time you have been bothered by your bloating problem?

① All the time ② Most of the time ③ Quite Often ④ Sometimes

⑤ A Little of the Time ⑥ Hardly Any ⑦ Never

2. In the past 2 wk, how much time did you feel fatigued or tired?

① All the time ② Most of the time ③ Quite Often ④ Sometimes

⑤ A Little of the Time ⑥ Hardly Any ⑦ Never

3. In the past 2 wk, how much time have you experienced bodily pain?

① All the time ② Most of the time ③ Quite Often ④ Sometimes

⑤ A Little of the Time ⑥ Hardly Any ⑦ Never

4. In the past 2 wk, how often did you feel sleepy during the daytime?

① All the time ② Most of the time ③ Quite Often ④ Sometimes

⑤ A Little of the Time ⑥ Hardly Any ⑦ Never

5. In the past 2 wk, how much time did you have abdominal pain?

① All the time ② Most of the time ③ Quite Often ④ Sometimes

⑤ A Little of the Time ⑥ Hardly Any ⑦ Never

6. In the past 2 wk, how much time have you had a shortness of breath in your daily activities?

① All the time ② Most of the time ③ Quite Often ④ Sometimes

⑤ A Little of the Time ⑥ Hardly Any ⑦ Never

7. In the past 2 wk, how much time were you unable to eat as much as you want?

① All the time ② Most of the time ③ Quite Often ④ Sometimes

⑤ A Little of the Time ⑥ Hardly Any ⑦ Never

8. In the past 2 wk, how much time have you been bothered by the decreased physical energy?

① All the time ② Most of the time ③ Quite Often ④ Sometimes

⑤ A Little of the Time ⑥ Hardly Any ⑦ Never

9. In the past 2 wk, how often did you feel difficult to lift or carry heavy objects?

① All the time ② Most of the time ③ Quite Often ④ Sometimes

⑤ A Little of the Time ⑥ Hardly Any ⑦ Never

10. In the past 2 wk, how often did you feel anxious?

① All the time ② Most of the time ③ Quite Often ④ Sometimes

⑤ A Little of the Time ⑥ Hardly Any ⑦ Never

11. In the past 2 wk, how often did you find your energy level decreasing?

① All the time ② Most of the time ③ Quite Often ④ Sometimes

⑤ A Little of the Time ⑥ Hardly Any ⑦ Never

12. In the past 2 wk, how much time did you feel unhappy?

① All the time ② Most of the time ③ Quite Often ④ Sometimes

⑤ A Little of the Time ⑥ Hardly Any ⑦ Never

13. In the past 2 wk, how often did you feel drowsy?

① All the time ② Most of the time ③ Quite Often ④ Sometimes

⑤ A Little of the Time ⑥ Hardly Any ⑦ Never

14. In the past 2 wk, how much time have you been bothered by loss of appetite?

① All the time ② Most of the time ③ Quite Often ④ Sometimes

⑤ A Little of the Time ⑥ Hardly Any ⑦ Never

15. In the past 2 wk, how often did you become easy to get angry?

① All the time ② Most of the time ③ Quite Often ④ Sometimes

⑤ A Little of the Time ⑥ Hardly Any ⑦ Never

16. In the past 2 wk, how much time did you find it difficult to sleep at night?

① All the time ② Most of the time ③ Quite Often ④ Sometimes

⑤ A Little of the Time ⑥ Hardly Any ⑦ Never

17. In the past 2 wk, how much time have you been bothered by your abdominal discomfort?

① All the time ② Most of the time ③ Quite Often ④ Sometimes

⑤ A Little of the Time ⑥ Hardly Any ⑦ Never

18. In the past 2 wk, how much time did you worry that your liver disease will affect your family?

① All the time ② Most of the time ③ Quite Often ④ Sometimes

⑤ A Little of the Time ⑥ Hardly Any ⑦ Never

19. In the past 2 wk, how much time did your emotions swing?

① All the time ② Most of the time ③ Quite Often ④ Sometimes

⑤ A Little of the Time ⑥ Hardly Any ⑦ Never

20. In the past 2 wk, how much time were you unable to sleep at night?

① All the time ② Most of the time ③ Quite Often ④ Sometimes

⑤ A Little of the Time ⑥ Hardly Any ⑦ Never

21. In the past 2 wk, how often did your muscle cramp?

① All the time ② Most of the time ③ Quite Often ④ Sometimes

⑤ A Little of the Time ⑥ Hardly Any ⑦ Never

22. In the past 2 wk, how much time did you worry that your symptoms will develop into a serious problem?

① All the time ② Most of the time ③ Quite Often ④ Sometimes

⑤ A Little of the Time ⑥ Hardly Any ⑦ Never

23. In the past 2 wk, how much time did you have dry mouth?

① All the time ② Most of the time ③ Quite Often ④ Sometimes

⑤ A Little of the Time ⑥ Hardly Any ⑦ Never

24. In the past 2 wk, how much time did you feel depressed?

① All the time ② Most of the time ③ Quite Often ④ Sometimes

⑤ A Little of the Time ⑥ Hardly Any ⑦ Never

25. In the past 2 wk, how much time did you worry that your health condition will deteriorate?

① All the time ② Most of the time ③ Quite Often ④ Sometimes

⑤ A Little of the Time ⑥ Hardly Any ⑦ Never

26. In the past 2 wk, how much time did you find it difficult to concentrate?

① All the time ② Most of the time ③ Quite Often ④ Sometimes

⑤ A Little of the Time ⑥ Hardly Any ⑦ Never

27 .In the past 2 wk, how much time have you been bothered by itchiness?

① All the time ② Most of the time ③ Quite Often ④ Sometimes

⑤ A Little of the Time ⑥ Hardly Any ⑦ Never

28. In the past 2 wk, how much time did you worry that your health condition will not get better?

① All the time ② Most of the time ③ Quite Often ④ Sometimes

⑤ A Little of the Time ⑥ Hardly Any ⑦ Never

1. In the past 2 wk, how much time have you worried that your economic reasons will impact on your disease treatment?

① All the time ② Most of the time ③ Quite Often ④ Sometimes

⑤ A Little of the Time ⑥ Hardly Any ⑦ Never

**III.Recent disease situation for the patient**

Fill the laboratory test findings of this time or the last time in the following space. Please fill in the findings you have received in secondary or tertiary hospital within 3 months, otherwise fill in the reviewed findings.

ALT(u/L) AST(g/L) TBIL(μmol/L)

PT(S) AFP(ng/mL) GLU(mmol/L)

HBsAg HBsAb HBeAg

HBeAb HBcAb HBV DNA(copies/L)
