# Supplementary material for: Development and Application of the Chinese (Mainland) Version of Chronic Liver Disease Questionnaire to Assess the Health-Related Quality of Life (HRQoL) in Patients with Chronic Hepatitis B
Source: PLoS One. 2016 Sep 15;11(9):e0162763. doi: 10.1371/journal.pone.0162763 (PMC5025145; doi:10.1371/journal.pone.0162763)
Supplement: S2 File — (DOCX) [file pone.0162763.s002.docx]

**The Original CLDQ**

This questionnaire is designed to find out how you have been feeling during the last 2 wk.

You will be asked about you symptoms related to your liver disease, how you have been affected in doing activities, and how your mood has been.

Please complete all of questions and select only one response for each question.

1 How much of the time during the last 2 wk have you been troubled by a feeling of abdominal bloating?

All of the time

Most of the time

A good bit of the time

Some of the time

A little of the time

Hardly any of the time

None of the time

2 How much of the time have you been tired or fatigued during the last 2 wk?

3 How much of the time during the last 2 wk have you experienced bodily pain?

4 How often during the last 2 wk have you felt sleepy during the day?

5 How much of the time during the last 2 wk have you experienced abdominal pain?

6 How much of the time during the last 2 wk has shortness of breath been for you in your daily activities?

7 How much of the time during the last 2 wk have you not been able to eat as much as you would like?

8 How much of the time in the last 2 wk have you been bothered by having decreased strength?

9 How often during the last 2 wk have you had trouble lifting or carrying heavy objects?

10 How often during the last 2 wk have you felt anxious?

11 How often during the last 2 wk have you felt a decreased level of energy?

12 How much of the time during the last 2 wk have you felt unhappy?

13 How often during the last 2 wk have you felt drowsy?

14 How much of the time during the last 2 wk have you been bothered by a limitation of your diet?

15 How often during the last 2 wk have you been irritable?

16 How much of the time during the last 2 wk have you had difficulty sleeping at night?

17 How much of the time during the last 2 wk have you been troubled by a feeling of abdominal discomfort?

18 How much of the time during the last 2 wk have you been worried about the impact your liver disease has on your family?

19 How much of the time during the last 2 wk have you had mood swings?

20 How much of the time during the last 2 wk have you been unable to fall asleep at night?

21 How often during the last 2 wk have you had muscle cramps?

22 How much of the time during the last 2 wk have you been worried that your symptoms will develop into major problems?

23 How much of the time during the last 2 wk have you had a dry mouth?

24 How much of the time during the last 2 wk have you felt depressed?

25 How much of the time during the last 2 wk have you been worried about your condition getting worse?

26 How much of the time during the last 2 wk have you had problems concentration?

27 How much of the time have you been troubled by itching during the last 2 wk?

28 How much of the time during the last 2 wk have you been worried about never feeling any better?

29 How much of the time during the last 2 wk have you been concerned about the availability of a liver if you need a liver transplant?

**The Chinese(Mainland) CLDQ**

The purpose of this section is to understand how you felt in the past 2 wk. Please answer all questions. You can only choose one answer for each question.

1. In the past 2 wk, how much time you have been bothered by your bloating problem?

All of the time

Most of the time

A good bit of the time

Some of the time

A little of the time

Hardly any of the time

None of the time

2. In the past 2 wk, how much time did you feel fatigued or tired?

3. In the past 2 wk, how much time have you experienced bodily pain?

4. In the past 2 wk, how often did you feel sleepy during the daytime?

5. In the past 2 wk, how much time did you have abdominal pain?

6. In the past 2 wk, how much time have you had a shortness of breath in your daily activities?

7. In the past 2 wk, how much time were you unable to eat as much as you want?

8. In the past 2 wk, how much time have you been bothered by the decreased physical energy?

9. In the past 2 wk, how often did you feel difficult to lift or carry heavy objects?

10. In the past 2 wk, how often did you feel anxious?

11. In the past 2 wk, how often did you find your energy level decreasing?

12. In the past 2 wk, how much time did you feel unhappy?

13. In the past 2 wk, how often did you feel drowsy?

14. In the past 2 wk, how much time have you been bothered by loss of appetite?

15. In the past 2 wk, how often did you become easy to get angry?

16. In the past 2 wk, how much time did you find it difficult to sleep at night?

17. In the past 2 wk, how much time have you been bothered by your abdominal discomfort?

18. In the past 2 wk, how much time did you worry that your liver disease will affect your family?

19. In the past 2 wk, how much time did your emotions swing?

20. In the past 2 wk, how much time were you unable to sleep at night?

21. In the past 2 wk, how often did your muscle cramp?

22. In the past 2 wk, how much time did you worry that your symptoms will develop into a serious problem?

23. In the past 2 wk, how much time did you have dry mouth?

1. In the past 2 wk, how much time did you feel depressed?

25. In the past 2 wk, how much time did you worry that your health condition will deteriorate?

26. In the past 2 wk, how much time did you find it difficult to concentrate?

27 .In the past 2 wk, how much time have you been bothered by itchiness?

28. In the past 2 wk, how much time did you worry that your health condition will not get better?

1. In the past 2 wk, how much time have you worried that your economic reasons will impact on your disease treatment?
