# Supplementary material for: Development and Application of the Chinese (Mainland) Version of Chronic Liver Disease Questionnaire to Assess the Health-Related Quality of Life (HRQoL) in Patients with Chronic Hepatitis B
Source: PLoS One. 2016 Sep 15;11(9):e0162763. doi: 10.1371/journal.pone.0162763 (PMC5025145; doi:10.1371/journal.pone.0162763)
Supplement: S1 File — (ZIP) [file pone.0162763.s001.zip › ╡╪╠│╥╜╘║║═╙╙░▓╥╜╘║┬2╨╘╕╬▓í╬╩╛φ.doc]

**慢性肝病问卷**

您好！为更好地服务于患者，提高医疗服务质量，我们在佑安医院和地坛医院的门诊对就诊患者进行近两周的生活质量和患病情况调查。本次调查不记名，您所填信息仅供研究和改善服务质量之用，您的个人信息不会暴露。请在您所选的答案序号上划“√”。非常感谢您的支持与配合！

首都医科大学

2011年4月8日

**I. 患者基本情况**

N1 您的性别：① 男	② 女

N2 您的年龄：	周岁

N3 您的文化程度：①大学及以上 ②高中及同等学历 ③初中 ④小学 ⑤文盲

N4 您所从事的职业：①专业技术人员 ②工人 ③农业劳动者	④学生 ⑤离退休

⑥家务或待业	⑦商业服务业 ⑧其他

N5您的医疗费用类型:	①社会医疗保险	②公费	③新农合	④自费	⑤商业医疗保险	⑥其他

N6 您的婚姻状态：①在婚 ②未婚 ③离婚或丧偶

N7 您被确诊为乙肝的时间是：	年	月 ( 总计	年 )

N8 您现在是否合并肝硬化（医生确诊）？ ①否	②是（确诊时间：	年	月）

N9 自患病以来，医生是否告诉您合并有肝硬化的并发症？	①是	②否**（跳至N11）**

N10您曾患有肝硬化并发症的类型是属于以下哪种疾病？（可多选）

1. 腹水（①4周内消退 ②4周以上消退或一直存在） ②腹腔感染（如腹膜炎，胆道感染等）

③上消化道出血	④肝性脑病（①Ⅰ期 ②Ⅱ期 ③Ⅲ期 ④Ⅳ期） ⑤电解质紊乱	⑥其他

N11 您目前的疾病状态属于以下哪个类型？

①慢性乙肝（a 静止性	b 活动性）持续时间：	月

②慢乙肝肝硬化（a 代偿期	b 失代偿期）持续时间：	月

③肝癌 持续时间：	月

N12 您最近一个月是否饮酒？	①是	②否

N13　您患有其他慢病的情况：

①糖尿病 ②高血压 ③冠心病 ④脑卒中	⑤骨质疏松 ⑥其他	⑦无

N14 患病后，您是否按医生的医嘱定期到专科医院就诊？ ①是	②否

N15 自患病后,您是否使用过抗病毒药物治疗：①是	②否	③不知道

N16 最近一年来您工作的情况：

①一直坚持工作 ②因病休假3-6个月 ③因病休假6个月以上 ④自生病后没上班 ⑤退休

N17 您去年1年中因为慢性肝病所花的医疗总费用大约是多少（**不含交通费**）？

①少于1千元	②1～2千之间	③2～3千之间	④4～5千之间

⑤5千～1万元	⑥1～2万元之间	⑦超过2万元

N18去年您家庭的总收入大约是 　　 　元，家庭人均收入大约是	元/人。

N19 您的家人中是否有人患肝炎？①有	②无 ③不清楚

N20 过去一年中，您是否因为慢性肝病去看过社区医生/全科医生？	①是	②否

N21 如果社区/全科医生可为您提供慢乙肝随访服务，您是否愿意接受这种服务？	①是	②否

**II. 患者生活质量的测评** （以下问题用于评测您在**近两周**的总体感觉，请选择您认为最合适的答案。）

N1 近两周内，您是否出现过**腹胀**？

①总是；②大部分时间；③ 经常；④有时；⑤偶尔；⑥很少；⑦从来没有

N2 近两周内，您是否感到过**疲乏和劳累**？

①总是；②大部分时间；③ 经常；④有时；⑤偶尔；⑥很少；⑦从来没有

N3 近两周内，您身体上是否感到过**疼痛**？

①总是；②大部分时间；③ 经常；④有时；⑤偶尔；⑥很少；⑦从来没有

N4 近两周内，您在白天时是否觉得**想睡觉**？

①总是；②大部分时间；③ 经常；④有时；⑤偶尔；⑥很少；⑦从来没有

N5 近两周内，您是否出现过**腹痛**吗？

①总是；②大部分时间；③ 经常；④有时；⑤偶尔；⑥很少；⑦从来没有

N6 近两周内，您日常活动时是否感到过**气促、气短**吗？

①总是；②大部分时间；③ 经常；④有时；⑤偶尔；⑥很少；⑦从来没有

N7 近两周内，您是否感到过**食欲不振**、不想吃东西？

①总是；②大部分时间；③ 经常；④有时；⑤偶尔；⑥很少；⑦从来没有

N8 近两周内，您是否为您的**体能下降**担心？

①总是；②大部分时间；③ 经常；④有时；⑤偶尔；⑥很少；⑦从来没有

N9 近两周内，您提重物时或爬楼梯时感到**吃力**吗？

①总是；②大部分时间；③ 经常；④有时；⑤偶尔；⑥很少；⑦从来没有

N10 近两周内，您是否感到**焦躁不安**？

①总是；②大部分时间；③ 经常；④有时；⑤偶尔；⑥很少；⑦从来没有

N11 近两周内，您是否感到过你的**体力不足**？

①总是；②大部分时间；③ 经常；④有时；⑤偶尔；⑥很少；⑦从来没有

N12 近两周内，您是否感到**不高兴**？

①总是；②大部分时间；③ 经常；④有时；⑤偶尔；⑥很少；⑦从来没有

N13 近两周内，您是否感到**很困**？

①总是；②大部分时间；③ 经常；④有时；⑤偶尔；⑥很少；⑦从来没有

N14 近两周内，您是否为您的**食欲不振感到烦恼**？

①总是；②大部分时间；③ 经常；④有时；⑤偶尔；⑥很少；⑦从来没有

N15 近两周内，您是否**发过脾气**？

①总是；②大部分时间；③ 经常；④有时；⑤偶尔；⑥很少；⑦从来没有

N16 近两周内，您是否在晚上出现过**入睡困难**？

①总是；②大部分时间；③ 经常；④有时；⑤偶尔；⑥很少；⑦从来没有

N17 近两周内，您是否因为**腹部不适**感到过困扰？

①总是；②大部分时间；③ 经常；④有时；⑤偶尔；⑥很少；⑦从来没有

N18 近两周内，您是否担心您的肝病会对你的**家庭产生影响**？

①总是；②大部分时间；③ 经常；④有时；⑤偶尔；⑥很少；⑦从来没有

N19 近两周内，您的**情绪**是否**不稳定**、时好时坏？

①总是；②大部分时间；③ 经常；④有时；⑤偶尔；⑥很少；⑦从来没有

N20 近两周内，您晚上是否有过**失眠**？

①总是；②大部分时间；③ 经常；④有时；⑤偶尔；⑥很少；⑦从来没有

N21 近两周内，您是否出现**肌肉抽筋**？

①总是；②大部分时间；③ 经常；④有时；⑤偶尔；⑥很少；⑦从来没有

N22 近两周内，您是否担心您的**症状会进展**到一个很严重情况？

①总是；②大部分时间；③ 经常；④有时；⑤偶尔；⑥很少；⑦从来没有

N23 近两周内，您是否出现过**口干**？

①总是；②大部分时间；③ 经常；④有时；⑤偶尔；⑥很少；⑦从来没有

N24 近两周内，您是否出现过**情绪低落**？

①总是；②大部分时间；③ 经常；④有时；⑤偶尔；⑥很少；⑦从来没有

N25 近两周内，您是否担心您的**病情会恶化**？

①总是；②大部分时间；③ 经常；④有时；⑤偶尔；⑥很少；⑦从来没有

N26 近两周内，您是否感到很难**集中精力**？

①总是；②大部分时间；③ 经常；④有时；⑤偶尔；⑥很少；⑦从来没有

N27 近两周内，您是否出现过**皮肤瘙痒**的问题？

①总是；②大部分时间；③ 经常；④有时；⑤偶尔；⑥很少；⑦从来没有

N28 近两周内，您是否担心您的**病情不会好转**？

①总是；②大部分时间；③ 经常；④有时；⑤偶尔；⑥很少；⑦从来没有

N29 近两周内，您是否担心因为**经济困难**影响您的疾病治疗？

①总是；②大部分时间；③ 经常；④有时；⑤偶尔；⑥很少；⑦从来没有

**III.您最近的疾病状况**

您本次或最近一次实验检查检查结果：填写近3月内检查结果(二级以上医院)，否则填写复查结果。

ALT(u/L)	AST(g/L)	TBIL(μmol/L)

PT(S)	AFP(ng/mL)	GLU(mmol/L)

HBsAg	HBsAb	HBeAg

HBeAb	HBcAb	HBV DNA(copies/L)
